# Building a resilient health system for universal health coverage and health security: a systematic review

**DOI:** 10.1186/s41256-023-00340-z

**Published:** 2024-01-04

**Authors:** Ayal Debie, Adane Nigusie, Dereje Gedle, Resham B. Khatri, Yibeltal Assefa

**Affiliations:** 1 Departement of Health Systems and Policy, Institute of Public Health, University of Gondar, Gondar, Ethiopia; 2 Departement of Health Education and Behavioral Sciences, Institute of Public Health, University of Gondar, Gondar, Ethiopia; 3 School of Public Health, The University of Queensland, Brisbane, Australia; 4 College of Medicine and Public Health, Flinders University, Adelaide, Australia

**Keywords:** Health system, Health security, Resilient, Universal health coverage

## Abstract

**Background:**

Resilient health system (RHS) is crucial to achieving universal health coverage (UHC) and health security. However, little is known about strategies towards RHS to improve UHC and health security. This systematic review aims to synthesise the literature to understand approaches to build RHS toward UHC and health security.

**Methods:**

A systematic search was conducted including studies published from 01 January 2000 to 31 December 2021. Studies were searched in three databases (PubMed, Embase, and Scopus) using search terms under four domains: resilience, health system, universal health coverage, and health security. We critically appraised articles using Rees and colleagues’ quality appraisal checklist to assess the quality of papers. A systematic narrative synthesis was conducted to analyse and synthesise the data using the World Health Organization’s health systems building block framework.

**Results:**

A total of 57 articles were included in the final review. Context-based redistribution of health workers, task-shifting policy, and results-based health financing policy helped to build RHS. High political commitment, community-based response planning, and multi-sectorial collaboration were critical to realising UHC and health security. On the contrary, lack of access, non-responsive, inequitable healthcare services, poor surveillance, weak leadership, and income inequalities were the constraints to achieving UHC and health security. In addition, the lack of basic healthcare infrastructures, inadequately skilled health workforces, absence of clear government policy, lack of clarity of stakeholder roles, and uneven distribution of health facilities and health workers were the challenges to achieving UHC and health security.

**Conclusions:**

Advanced healthcare infrastructures and adequate number of healthcare workers are essential to achieving UHC and health security. However, they are not alone adequate to protect the health system from potential failure. Context-specific redistribution of health workers, task-shifting, result-based health financing policies, and integrated and multi-sectoral approaches, based on the principles of primary health care, are necessary for building RHS toward UHC and health security.

**Supplementary Information:**

The online version contains supplementary material available at 10.1186/s41256-023-00340-z.

## Background

Resilient health system (RHS) is essential to ensure universal health coverage (UHC) and health security. It is about the health system’s preparedness and response to severe and acute shocks, and how the system can absorb, adapt and transform to cope with such changes [1, 2]. Resilient health system reflects the ability to continue service delivery despite extraordinary shocks to achieving UHC [3]. A study in Nepal showed that adoption of coexistence strategy on the continuation of the international community on strengthening the health sector with the principle of “do-no-harm” and impartiality at the time of conflicts improve the health outcomes [4].

In 2015, the United Nations (UN) General Assembly adopted a new development agenda aiming to transform the world by achieving the Sustainable Development Goals (SDGs) by 2030 [5], based on the lessons from the Millennium Development Goals (MDGs) [6]. The SDGs seek to tackle the “unfinished business” of the MDGs era and recognise that health is a major contributor and beneficiary of sustainable development policies [7]. One of the 17 goals has been devoted specifically to health: “ensure healthy lives and promote well-being for all ages” [6]. All UN Member States have agreed to achieve UHC (target 3.8) by 2030, as part of the SDGs [8]. The 2030 UHC target was intended to reach at least 80% for the UHC service coverage index and 100% for financial protection [9]. Universal health coverage is achieved when everyone has access to essential healthcare services without financial hardship associated with paying for care [10].

Universal health coverage and health security are two sides of the same coin. They are interconnected and complementary goals that require strong health systems and public health infrastructure to ensure that everyone has access to essential health services [11]. Universal health coverage and health security require an integrated and multi-sectorial system strengthening to provide quality and equitable healthcare services across populations [12].

A resilient health system provides the foundation for both [11]. Strengthening the World Health Organisation’s (WHO’s) six health system building blocks, including service delivery, health workforce, health information systems, health financing, leadership and governance, and access to essential medicines and infrastructures are essential to achieve UHC and health security [13]. The 13th WHO programme is structured in three interconnected strategic priorities to ensure SDG-3 including: achieving UHC, addressing health emergencies, and promoting healthier populations [14].

In the World Health Organisation (WHO) European Region, health security emphasises on the analysis of infectious diseases, natural and human-made disasters, conflicts and complex emergencies, and potential future challenges from global changes, particularly climate change [15]. Health security is also considered as the activities required, both proactive and reactive, to minimise the danger and impact of acute public health events that endanger people’s health across geographical regions and international boundaries [16]. The links between health system and health security have started to emerge in several national strategic plans and global initiatives, such as the Global Health Security Agenda (GHSA) and One Health, which aim to better facilitate the implementation of the International Health Regulations (IHR) [17]. The aim of IHR is to prevent, detect, and respond to the international spread of disease in effective and efficient manner [18]. The GHSA also help countries to build their capacity to prevent, detect, and respond to infectious disease threats [19].

Although almost all nations are progressing towards UHC, the advancement in low and low-middle income countries (LLMICs) is slow [20]. This is because the ethos and organisations of many health systems are more suitable for yesterday’s disease burden than tomorrow [21, 22]. Health systems of various nations faced numerous shocks, including public health, social, economic and political crises associated with COVID-19 [23]. The COVID -19 pandemic has made an unprecedented impact on the international community and exposed the vulnerabilities of the present global health architecture [24]. The COVID-19 pandemic is a perfect reminder that countries, individually and collectively, require a strong RHS now more than ever; however, there was no adequate evidence on the strategies toward RHS to improving UHC and health security. Thus, this study can inform the global health community on the lessons of RHS and its applications to UHC in pandemic and beyond. This review generally aimed to address the following research questions: 1) What are the existing evidence on the impact of RHS for UHC and health security? 2) What are the essential elements and characteristics of RHS for UHC and health security as per the WHO building blocks? and 3) What examples exist to demonstrate on how to build RHS core components for UHC and health security?

## Methods

### Registration and search strategy

This review was conducted and reported following enhancing transparency in reporting the synthesis of qualitative research (ENTERQ). Following ENTERQ guidelines, the systematic review was registered with the international prospective register of systematic reviews (PROSPERO) on 02 January 2022 with registration: CRD42020210471. Studies were searched in three databases (PubMed, Embase and Scopus) using search terms of under four broader domains, including resilience, health system, universal health coverage, and health security. Additional literatures were identified by searching in Google and Google Scholar. The search strategies were built using the four domains of search terms, and “Title/Abstract” by linking “AND” and “OR” Boolean operator terms as appropriate (Additional file 1). We also used the ENTERQ checklist for reporting the articles (Additional file 2).

### Inclusion and exclusion criteria

All articles in relation to RHS towards UHC and health security were included in the review. Inclusion criteria were articles written in the English language published from 01 January 2000 to 31 December 2021. Qualitative, quantitative, and mixed methods studies were eligible for inclusion. Exclusion criteria were perspectives, commentary, expert’s opinion, conference papers, debates, conference reports, letters to the editor, and editorials. We presented this paper as a narrative review, following some components of the preferred reporting of systematic review and meta-analysis (PRISMA) guideline for scoping review (Additional file 3).

### Selection process

The primary author (AD) imported all retrieved articles into the Endnote library to remove duplicates. After removing the duplicates, three authors (AD, AN and DG) independently screened the articles by title and abstract based on inclusion criteria. The senior authors (RBK and YA) mediated the discrepancies between the three reviewers through discussion. Finally, we retained and reviewed the full texts of all relevant studies for final data synthesis.

### Data extraction and framework for synthesis

We used the Rees and colleagues’ appraisal instrument as a guiding tool to appraise the quality of included articles in the review [25]. The quality appraisal instrument is a comprehensive tool designed to assess the quality, rigor of research studies, covers key aspects of research design, data collection, analysis, and reporting. This includes rigour in sampling, rigour in data collection, rigour in data analysis, findings supported by the data, breadth and depth of findings, extent of the study privilege perspectives, reliability or trustworthiness, and usefulness[25]. A template was developed to extract relevant data from each eligible study. After reading the selected studies, key findings were extracted into the template, including information about the first author, year of publication, type of article, study design, and key summary findings. Three independent reviewers (AD, AN and DG) extracted the data. The senior authors (RBK and YA) verified the extracted information. The successes and challenges of RHS for UHC and health security were extracted using health system building blocks.

We analysed the findings using the WHO health system building blocks, including service delivery, health workforce, health information systems, medicines and infrastructures, healthcare financing, and leadership and governance [13]. We analysed the key challenges and successes of RHS for UHC and health security using the WHO health system frameworks. Framework analysis provides a systematic approach to analysing large amounts of textual data using pre-determined framework components. This allows the analyst and those commissioning the research to move between multiple layers of abstraction without losing sight of raw data [26].

## Results

### Search results

**Fig. 1:**
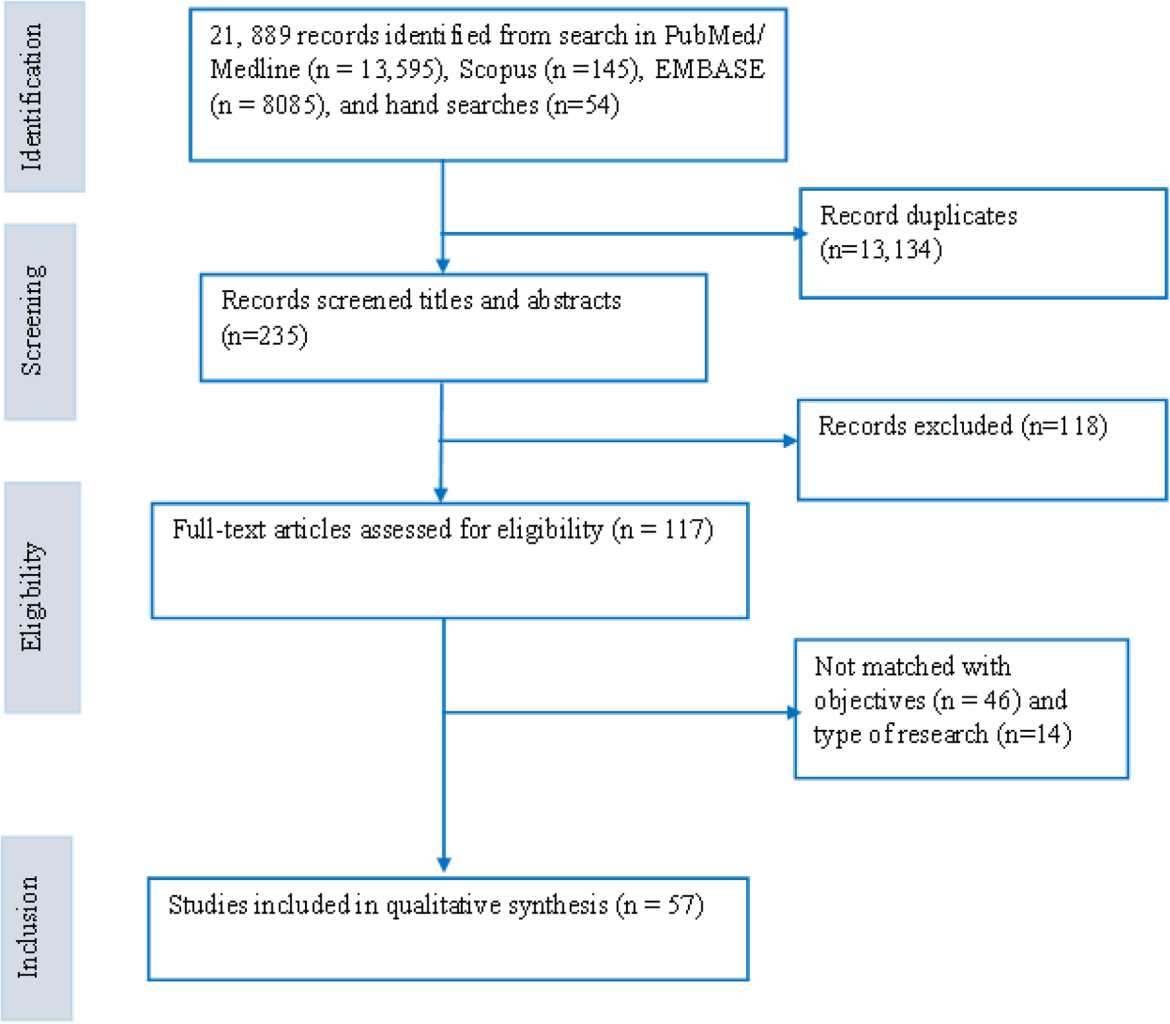
ENTREQ flow diagram for the articles included in the review

A total of 21,889 records were identified in the initial literature search. After removing 13,134 duplicates, 235 articles were screened by titles and abstracts, and 118 were excluded. Next, 117 studies were reviewed using the full texts, and finally, 57 articles met the inclusion criteria and were analyzed in the systematic review (Fig. 1). Of these, 32 articles were primary studies, and 25 articles investigated the application of RHS on UHC. In addition, nine articles explained RHS's implications on UHC and health security. Of these, approximately one-third (19 articles) were conducted in various African nations, while 19 articles were from Asian countries. The remaining articles were from other parts of the world. The study also reviewed articles on various aspects of health system building blocks, including health service delivery, health workforce, health information systems, health financing, leadership/governance, and medicines and infrastructure. The number of articles reviewed for each aspect were 17, 9, 10, 13, 22, and 10, respectively.

### Successes and challenges of RHS

This review used the six-health system building blocks to achieve UHC and health security. These include service delivery, health workforce, health information system, health financing, medicines, diagnostics and infrastructures, and leadership and governance (Table 1).

**Table 1:** Resilient health system for UHC and health security, 2022

Blocks	Universal health coverage		Health security	
	Successes	Challenges	Successes	Challenges
Service delivery	Good service delivery was required to provide comprehensive, accessible, continuous, effective, safe, timely, person-centered, and coordinated healthcare services with full accountability and minimum wastages [27]	An inward migration or mass casualty incidents compromised the quality of services and increased deaths attributed to delays in treatment [30]	Promote operational integration between health service continuity and emergency response through proactive planning across all income nations reduced health services disruptions during emergencies [29]	A combination of public health security threats from new and reemerging infectious diseases, and intentional dissemination of chemical or biological substances were the challenges to ensure health security [34]
	Continuation of healthcare service delivery in the face of extraordinary shocks facilitated UHC progress [3]	Lack of access to basic primary care, laboratories, and shortage of existing hospital infrastructure to isolate and treat infected people fueled the epidemic's spread during Ebola outbreak [31]		Ebola outbreak jeopardised and disrupted health service delivery in Liberia. This put the lives of many children at risk associated with the lack of treatment for common childhood illnesses [35, 36]
	Increased inputs led to improve service delivery and access to services [13]	Lack of basic health care infrastructure, well-trained personnel, and essential medical supplies affected healthcare access [32]		Climate change was increasing threats and leads to changes in the frequency, intensity, spatial extent, duration, timing of extreme weather and climate events [39]
	Transportation, communication infrastructures, capacity building, referral systems, intersectoral actions, and electronic healthcare platforms had an impact on healthcare services [28]	Uneven distribution of health facilities and poor public–private service level agreements led to geographical inequities and financial protection [33]		Inadequate primary care system capacity on provision of responsive health services to storm and flood-related health problems was also one of the challenges [41]
	Community resources, cohesion, and physical accesses were significant assets to improve service utilisation and quality [30]	Exclusion from work due to health problems can easily result in economic impoverishment and inequitable healthcare access [37]		
		Socially excluded population groups received health services from a dysfunctional publicly provided health system in Guatemala and Peru, which undermines the progress towards UHC [38]		
		Extreme weather events caused an increase in disease prevalence, such as malaria and other vector-borne diseases, malnutrition, food insecurity and food-borne diseases are notably occurring impacts in drought, floods, and extreme temperatures affected countries [40]		
Health workforce	A well-performing health workforce helped to provide responsive, fair and efficient health services to achieve the best health outcomes [27]	Low perception of risks by tourists/ pilgrims, ineffective training, poor control of risk factors for infectious diseases and shortages of infrastructures were the challenges to combat such contagious diseases [47]	An ad hoc redistribution of health workers to address shortages during acute shock had a knock-on effect on health services [43]	Distribution of health workers across the country was often inadequate to meet the unexpected needs of an acute crisis [43]
	Task-shifting and strengthening cross-site mentorship, learning, coordination, and the referral pathway assisted building RHS [42]	Healthcare workers practices to effectively manage pandemics such as COVID-19 were constrained by individual factors such as education, residence, location of work station, hygiene promotion, and social distance management [42]	Training on disaster preparedness and management and rewarding package increased willingness of healthcare workers to participate during disaster management [45]	Lack of skilled health workforce was a major obstacle to contain an outbreak and deaths attributed to delays in treatment [35]
			Monitoring and evaluating of frontline health levels about their preparedness against public health emergency threats was helpful for early detection and control of health threats [46]	Patient assessments by non-indigenous health workers during an emergency was the challenges on early identification and management of the acute events [30]
			Ensuring staff distribution, training, and introducing rewarding packages were essential to control the epidemics [44]	
Health information system	A well-functioning health information system (HIS) ensured the production, analysis, dissemination and use of reliable and timely information on health determinants, health systems performance and health status [27]	Poor data management, failure to carry out systematic risk assessments, and risk communications were responsible for misleading media reports, influencing decisions [51]	Building novel surveillance systems improved clinical care and health system preparedness to tackle health threats [48]	Lack of awareness, resources and insufficient electronic reporting system were observed [53]
	Building accountability, knowledge culture management, and evidence through regular data quality audits contributed to strengthening health management information systems [44]		Flexible automation and data processing were crucial for quick and quality surveillance [49]	Misinformation on risk and transmission of the outbreak. Eg, misinformation during Ebola outbreak affected most communities to put measures [54]
			Increasing the health system's capacity to process and communicate test results rapidly was a key measure in response to the pandemic [51]	Poor surveillance and late timing of responses [52]
			An integrated disease surveillance and laboratory evaluation generated information to minimise inefficiencies of disease-specific surveillance silos [50]	People approached traditional healers and lack knowledge on modern treatment [35]
Medical products, diagnostics and infrastructures	A well-functioning health system ensured equitable access to essential medical products, vaccines and technologies to assure quality, safety, efficacy and cost-effective healthcare services to the users [27]	High patient load beyond the health facility’s capacity exposed to lack of health infrastructures [30]	Rapid, cost-effective, sensitive, and specific diagnostic tests were the key factors that facilitate outbreak detection and reporting [53]	Lack of diagnostic facilities and surveillance severely hampered to contain the efforts of public health threats. For instance, the long diagnostic services processes often complicated the response strategies and hampered contact tracing of Ebola [32]
	Strengthening local preparedness and planning, manufacturing, coordination of public–private initiatives, and trainings in LMICs was important to attain UHC in context of health crisis [55]	War related destruction of facilities compromised the health system’s hardware (health facilities and supplies). Decreased healthcare services in relation with physical healthcare destructions in Syria [3]	Inauguration of the national center for infectious disease purpose-built infrastructures integrated with clinical, laboratory and epidemiologic functions was vital to contain health emergencies. For instance, after the severe acute respiratory syndrome (SARS) outbreak, Singapore built a specialised infectious disease center for outbreak management [56]	Lack of medical supplies, PPE, and electricity increased the rate of Ebola infections [35]
		The critical challenge was the lack of a specific budget for medicine procurement, absence of emergency stockpile, and no proper means for medicine transportation [57]	An emergency plan considering list of medicines, adaptable mobile health care units and system for mobilisation of health professionals were crucial to curb health emergencies [57]	Longer lead times of medicine outlets associated with poor inter-country transportation, bureaucratic bottlenecks and lack of emergency readiness were the challenges in Namibia [55]
		Inadequate health professionals and essential logistics inhibited to handle potential maternal mortality. For instance, inadequate essential logistics such as blood, oxygen cylinders, ergometrine and sulphadoxine paramita mine in Ghana were the causes of low preparedness to control maternal mortality [58]		
Health financing	Good HCF system raised adequate funds for health to protect people from financial catastrophe to access health [27]	Low affordability of medical costs at private facilities and transport costs were the barriers to universal financial protection [33]		Lack of adequate funds invested in health system infrastructure contributed to poor management of an outbreak [35]
	Health financing system under public control improved compliance, sustainability and equity [59]	Performance-based financing without accompanying access to incentives for poor people was unlikely to improve equity [64]		Health sector requires additional financial support to address the demand for health services during health emergencies [30]
	Integration of financing mechanisms with high income, risk cross-subsidies, and reduced out-of-pocket payments maximised risk pools and resource allocation mechanisms to achieve UHC [60]	Health equity advancement challenges were securing dedicated funding to support transformative learning opportunities and build infrastructure [65]		Levels of funding from international donors were erratic and far below the amounts required to meet the health needs of the refugees during crisis [66]
	UHC substantially improved human security by financial security [61]	People could not trade their commodities because of the fear of attacks which exposed users to a lack of finances [30]		Falling in financial access to health services resulted in political demonstrations and violent unrest [67]
	Redistribution of income improved equity in health care service delivery [63]			
	Universal health coverage indicators were positively associated with the gross domestic product (GDP) per capita and the share of health spending channels [62]			
Leadership and Governance	Good health governance ensured strategic policy frameworks combined with effective oversight, coalition building, appropriate regulations, system-design, and accountability [27]	Absence of clear government policy for the delivery channels and financial coverage mechanism led to fragmentation and poor health system response to the refugee crisis in Syria [66]	Centralised governance coordinates the national policy responses and facilitates the homogenous implementation of measures [70]	Outbreak governance was weak with a lack of clarity of stakeholder roles [52]
	Clear communication channels between the government and all sectors were vital to translate policy into intended actions [56]	Poor leadership at the national government level was the main reason for poor coordination and absence of a prompt response [35]	Building responsible leadership and social capital at community level were needed to address health shocks [54]	Working alone, the state had proven only partially effective, a situation exacerbated by the natural tendency within the public to ignore as irrelevant to themselves [73]
	Multisectoral and multilevel control activities required environmental, political, social, and medical inputs to be coordinated and productive [52]	Healthcare services significantly decreased concerning physical healthcare destructions by war in Syria [3]	Effective governance processes built strong partnerships for health and accountability to respond to emergencies [48]	Weak governance and decision-making processes, especially high bureaucracy, weak prevention culture and lack of coordination explained a substantial part of the rapid spread of the virus in French in the first wave of COVID-19 [74]
	Vertical and horizontal integration made the administrative system more transparent and acceptable to the people [69]		High-performance teams during pandemic required leadership resilience to achieve health sector goals [71]	It was difficult for the system to automatically adjust its structure to reduce uncertainty and ascertain the complex adaptive behavior when facing public health emergencies. As a result, community action lacked an effective control form and even appears as an overcorrection phenomenon to highlight the work or evade political responsibility [79]
	Community participation was an approach to co-learning, community-developed research perspectives, shared decision making, and local capacity building [65]		Coordination, rationalisation, and connection of pandemic planning across sectors and jurisdictions resulted better preparedness [68]	
	Community participation between stakeholders had a significant impact on the successful implementation of health program. For example, prevention of Japanese encephalitis/ acute encephalitis syndrome through community health workers/ volunteers [77]		Global community increased its investment in early warning and detection systems to enable action [72]	
	Moving away from a one-size fits-to all approach in guiding pandemic response, service delivery, political commitment, fair contribution and distribution of resources are helpful to speed up the path towards UHC [75]		Community engagement in health surveillance activities in Cambodia enabled early detection and collection of mortality data [78]	
	Village health volunteers in Thailand, Zanmi Lazante’s Community Health Program in Haiti, Agentes Polivalentes Elementares in Mozambique, Village Health Teams in Uganda, lady health workers in Pakistan, BRAC in Bangladesh, Family Health Program in Brazil, and Health Extension Program in Ethiopia are successful community-based models contributed immensely to achieve health development goals [76]		An overarching political will and well-integrated and locally grounded health system might be more resilient to external shocks [80]	
			During crisis, political leadership was critical to develop a response strategy and effective implementation [81]	
			Singapore’s dexterous political environment allowed the government to swiftly institute measures for COVID-19 containment [56]	

### Service delivery

Of the total reviewed articles, 17 described their healthcare service delivery findings. Good service delivery provides comprehensive and person-centered healthcare services with full accountability [27]. Continuation of healthcare service delivery in the face of extraordinary shocks facilitated UHC progress [3]. Studies reported that health service inputs, access to transportation, communication infrastructures, capacity building, referral systems, intersectoral actions, and electronic healthcare platforms could facilitate service delivery and improve access to health services [13, 28]. Operational integration between health service continuity and emergency response through proactive planning across all income nations reduced health services disruptions during emergencies [29]. Community resources, cohesion, and physical accesses were significant assets to improve service utilisation and quality [30].

An inward migration or mass casualty incidents compromised the quality of services and increased deaths attributed to delays in treatment [30]. In the Ebola crisis, the long-standing lack of access to basic primary health care to isolate and treat infected people fueled the epidemic’s spread resulting in a death toll [31]. Uneven health facilities distribution and lack of well-trained personnel and supplies led to geographical inequities and poor healthcare access [32, 33]. A combination of public health security threats, both new and reemerging infectious diseases, challenged ensuring health security [34]. For example, the health service delivery, mainly the lives of many children, was at risk associated with the lack of treatment for common childhood illnesses in Liberia during the Ebola outbreak [35, 36]. Exclusion from work due to health problems can easily result in economic impoverishment and inequitable healthcare access, which will undoubtedly worsen health status [37]. For instance, socially excluded population groups received health services from a dysfunctional publicly provided health system marked by gaps and often invisible barriers in Guatemala and Peru, which undermines the progress towards UHC [38]. The changes in frequency, intensity, spatial extent, duration, and timing of extreme weather and climate events were also exposed to health threats [39]. For example, extreme weather events caused an increase in disease prevalence, such as malaria and other vector-borne diseases, malnutrition, food insecurity and food-borne diseases [40]. Inadequate primary health care system capacity to provide responsive health services to storm and flood-related health problems was another challenge [41].

### Health workforce

In our review, nine articles reported their findings on the successes and challenges of health workforces towards UHC and health security. A well-performing health workforce provides responsive, fair and efficient health services to achieve the best health outcomes [27]. Task-shifting policy, ensuring accountability and ad hoc redistribution of health workers had a knock-on effect on health services delivery and building RHS [42–44]. Training on disaster preparedness and management, and rewarding packages, such as incentives and hazard allowance, facilitated healthcare workers willing to participate in disaster management [45]. Monitoring and evaluating frontline health workers levels of preparedness against public health emergency threats periodically by their higher-level hierarchy was crucial for early detection and control of health threats [46].

Lack of skilled and inadequate health workforce distribution was the major obstacle to containing an outbreak, and deaths were attributed to treatment delays [35, 43]. Low perception of risks by tourists/ pilgrims, ineffective training, poor control of risk factors, and shortages of infrastructures were the challenges in combating contagious diseases [47]. Healthcare workers’ practices on effective pandemic management, including corona-virus disease (COVID)-19 were constrained by individual factors, such as education, residence, work station location, hygiene promotion, and social distance management [42]. Patient assessments by non-indigenous health workers during an emergency were also barriers to early identification and management of acute health events [30].

### Health information system

In this review, ten articles described the contributions and challenges of health information on RHS to realise UHC and health security. A well-functioning HIS ensures the production, analysis, dissemination and use of reliable information for policy decisions [27]. Building accountability, knowledge culture management, and evidence through regular data quality audit strengthened health management information systems (HMIS) [44]. Integrated disease surveillance, flexible automation and data processing improved clinical care and health system preparedness to tackle health threats [48–50]. Strengthening the health system’s capacity was another key measure to rapidly process and communicate test results for pandemic responses [51].

Poor surveillance, late timing of responses and lack of triggers weakened the functionality of plans and exposed to a high burden of diseases [52]. Poor data management, misinformation on the risk and transmission, lack of awareness, resources and insufficient electronic reporting system were responsible for the spread of diseases [51, 53, 54]. For instance, misinformation during the Ebola outbreak affected most communities in putting measures in place to stop the spread of the virus [54]. People approached traditional healers who lacked knowledge on treating certain health shocks in modern medicine was the major problems in early responses [35].

### Medical products, diagnostics, and infrastructures

Of the reviewed articles, 10 reported their findings on the successes and constraints of medical products, diagnostics, and infrastructures to realise UHC and health security. Equitable access to essential medical products, vaccines and technologies to assure quality, safety, efficacy and cost-effective healthcare services to users was the attribute of a well-functioning health system [27]. To attain UHC, strengthening local preparedness, planning, manufacturing, and coordinating public–private initiatives and training in LMICs was important [55]. The key factors to facilitate early detection were the provision of rapid, cost-effective, sensitive, and specific diagnostic centers through the inauguration of national centers [53, 56]. Identifying emergency medicines, adaptable mobile health care units and systems for mobilisation of health professionals contributed to successful interventions to curb health emergencies [57].

High patient load, lack of diagnostics, destruction of health facilities and lack of specific funds for medicine procurement may compromise the health system’s hardware (health facilities and supplies) and contained public health threats [3, 30, 32, 57]. For instance, inadequate essential logistics such as blood, oxygen cylinders, ergometrine and sulphadoxine paramita mine in Ghana was the causes for low level of preparedness to control maternal mortality [58]. Shortages of medical supplies, personal protective equipment (PPE), and electricity increased the rate of Ebola infections during the outbreak [35]. Most medicine outlets experienced longer lead times associated with the poor inter-country transportation and limited manufacturing capacity, which were also Namibia's main challenges [55].

### Health financing

In this study, 13 articles described their findings on the contributions and limitations of healthcare financing to realise UHC and health security. A good health financing system raised adequate funds for health to ensure people can use needed services and be protected from financial catastrophes [27]. Under a publicly funded health financing system that fits well with values and population preferences improved compliance, sustainability, and equity [59]. An integrated financing mechanism through high income and risk cross-subsidies reduced reliance on OOP payments, maximises risk pools and resource allocation mechanisms facilitated to achieve UHC [60]. Universal health coverage can substantially improve human security through securing finances [61]. Universal health coverage indicators were also positively associated with the gross domestic product (GDP) per capita and the share of health spending channels [62]. Income redistribution improved equity in health care service delivery [63].

Lack of adequate funds and non-affordable medical costs were the main barriers to universal financial protection and poor management of an outbreak [33, 35]. In Burundi, for example, performance-based financing without accompanying access to incentives for the poor was the critical challenge to improve equity in health [64]. Health equity advancement challenges secured dedicated funds to support transformative learning opportunities and build infrastructures [65]. Because of causalities, the health sector requires additional financial support to address the increased demand for health services; however, movement restrictions limit people’s access to participate in gainful activities [30]. Low funds from international donors were erratic and far below the amounts required to meet the health needs at crisis time [66]. People could not trade their commodities because of the fear of attacks exposing service users to lack finances [30]. Falling in financial access to health services has resulted in political demonstrations and violent unrest [67].

### Leadership and governance

Our review found that 22 articles reported their findings on health system governance (HSG) to realise UHC and health security. Good HSG ensured strategic policy frameworks combined with effective oversight, coalition, appropriate regulations, system design and accountability [27]. Building strong partnerships, ensuring accountability, coordination, rationalisation, and connection of pandemic planning across sectors and jurisdictions resulted in better preparedness [48, 68]. Clear communication channels, multisectoral, and multilevel controls were essential to translate policy into actions [52, 56]. Vertical and horizontal integration, centralised governance, responsible leadership, and social capital at community level were needed to address health shocks and homogenous implementation of health interventions [54, 69, 70]. Fueling high-performing teams and increasing investment in early warning and detection systems required leadership resilience to enable action at all levels [71, 72].

Working alone the state had proven only partially effective, a situation exacerbated by the natural tendency within the public to ignore as irrelevant to themselves [73]. In addition, lack of clarity of stakeholder roles, poor leadership and absence of clear government policy for the delivery channels and financial coverage led to fragmentation and poor health system response [35, 52, 66]. For instance, weak governance and decision-making processes, such as high bureaucracy, low prevention culture, and lack of coordination between primary, social and hospital care providers, indicated virus’s rapid spread in the French population in the first wave of COVID-19 [74].

Moving away from a one-size fits-to all approach in guiding pandemic response, service delivery, political commitment, fair contribution and distribution of resources are helpful to speed up the path towards UHC [75]. For example, village health volunteers in Thailand, Zanmi Lazante’s Community Health Program in Haiti, Agentes Polivalentes Elementares in Mozambique, Village Health Teams in Uganda, lady health workers in Pakistan, BRAC in Bangladesh, Family Health Program in Brazil, and Health Extension Program in Ethiopia are successful community-based models contributed immensely to achieve health development goals [76]. In addition, community participation and coordination between different stakeholders significantly impact the prevention of encephalitis in Japan [77], and early detection of cases and collection of mortality data in Cambodia [78]. On the contrary, it was difficult for the system to automatically adjust its structure to reduce uncertainty and ascertain complex adaptive behaviour when facing public health emergencies [79].

With an overarching political will, well-integrated and locally grounded health system can be more resilient to external shocks [80]. Political leadership was critical during the crisis, which helped the government to develop a response strategy and effective implementation [81]. For instance, Singapore’s dexterous political environment allowed the government to institute measures to control COVID-19 swiftly [56]. On the other hand, political instability or war in Syria affected healthcare services by destroying physical health care infrastructures [3].

## Discussion

**Fig. 2:**
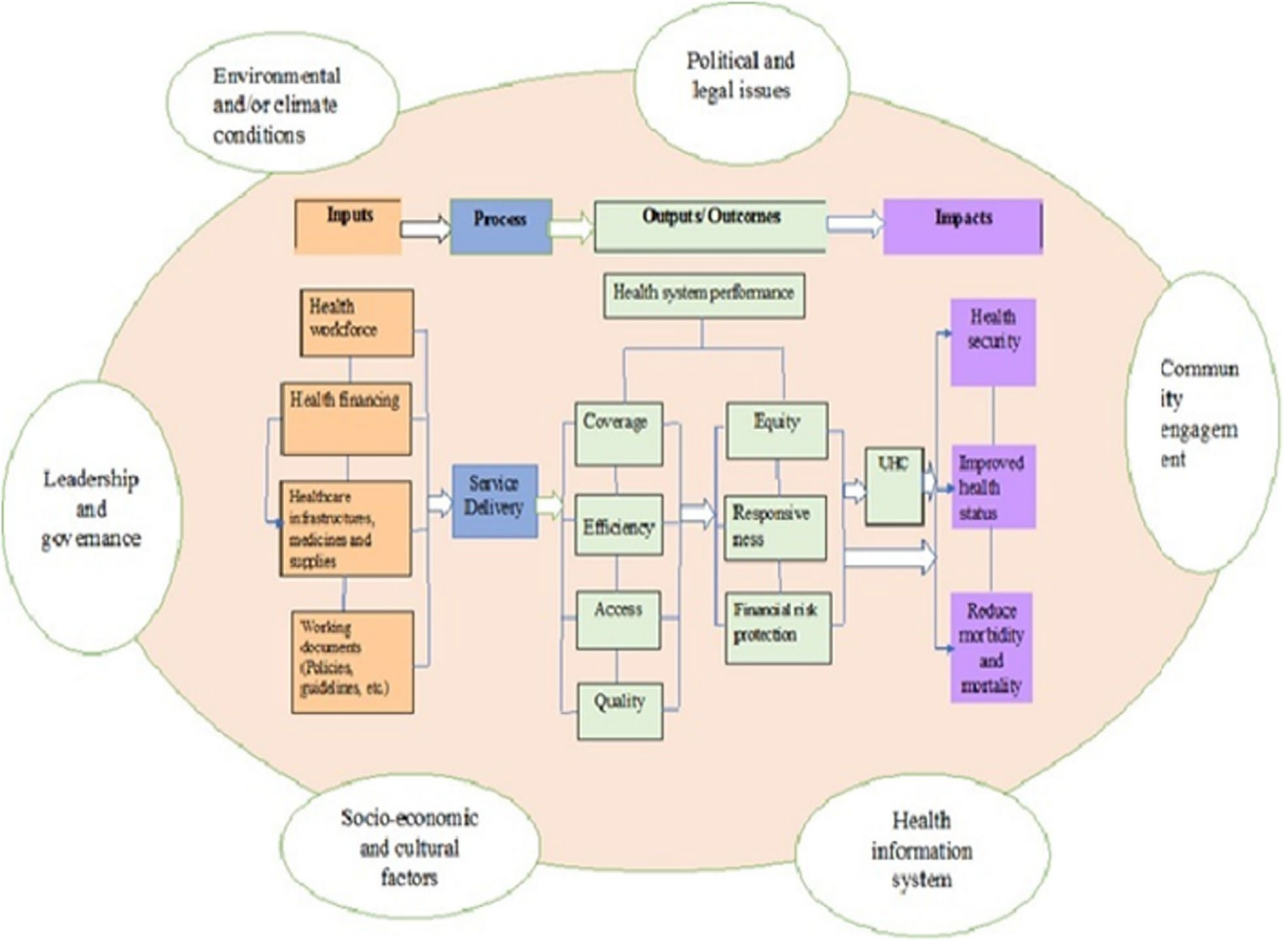
Resilient health system framework for UHC and health security

In this review, we developed a resilient health system framework that could assist countries in their endeavor toward universal health coverage and health security. The framework involves an integrated and multi-sectoral approach that considers the health system building blocks and contextual factors. The input components of the framework include health financing, health workforce, and infrastructure, while service delivery is the process component, and UHC and health security are the impact program components. The framework also considers health system performance attributes, such as access, equity, quality, safety, efficiency, sustainability, responsiveness, and financial risk protection. Additionally, the cross-cutting components of the framework are leadership and governance, health information systems (HIS), and contextual factors (e.g., political, environmental/climate, socioeconomic, and community engagement) that can affect the health system at any stage of the program components (Fig. 2).

We also indicated that RHS is critical to achieving UHC because it enables the provision of accessible, quality, and equitable health services, while also protecting people from financial risks associated with illness or injury. Such systems are built on strong primary healthcare services, effective governance and leadership, adequate financing, reliable health information systems, and a well-trained and motivated health workforce [42–44]. Resilient health systems are better equipped to deliver high-quality healthcare services to all people, including those who are marginalised or living in poverty. This, in turn, investing in RHS is essential for achieving UHC, promoting health equity, and building more sustainable and equitable societies. On the contrary, lack of healthcare access, skilled health workforces, and uneven distribution of health facilities and health workers [32, 33, 35, 43] were the challenges to achieving health sector goals.

Lack of access, non-responsive and inequitable healthcare services were the challenges to achieve UHC and health security [31–33]. Such challenges can be solved by primary health care approach which is an effective strategy to provide accessible, acceptable, equitable and affordable health services to achieve UHC [82, 83]. Community-based and differentiated service delivery models are also important platforms for improving healthcare delivery, access, outcomes, and to meet the specific needs and preferences of different groups of patients [84, 85]. Community-based service delivery model can bring healthcare services closer to where people live and work, and overcome barriers to healthcare access such as transportation, distance, and cost [84]. This service delivery model has also the potential to facilitate a more effective response during healthcare crises by minimising top-down approaches and maximising bottom-up strategies through empowering local communities to take ownership of their health and wellbeing [86]. Additionally, differentiated service delivery model can meet the specific needs and preferences of different groups of patients. For example, providing family planning services within HIV clinics helps women living with HIV to access both services at the same time [85]. Similarly, considering a health system away from a one-size-fits-all approach to healthcare delivery is essential in meeting the needs of diverse patient populations [87].

Lack of skilled and inadequate distribution of health workforces were another major obstacle to contain an outbreak and deaths attributed to delays in treatment [35, 43]. Conducting integrated supportive supervision, maintenance of human resource information systems, and national task shifting policy are important strategies that can help to address critical health workforce gaps and maldistribution [42, 44]. Healthcare workers' pre-service and in-service training opportunities are indeed key to providing quality care. Healthcare workers who receive adequate pre-service and in-service training are better equipped to provide quality care to patients, and also to adapt to new challenges and changing healthcare needs over time [88]. Training in disaster preparedness and offering rewarding packages can also play an essential role in enhancing the willingness of healthcare workers to participate in disaster management [45]. For instance, Kenya's Field Epidemiology and Laboratory Training Program (FELTP) has played a significant role in strengthening the capacity of healthcare workers to detect, document, respond, and report unusual health events [89]. In addition, monitoring frontline health levels is an essential part of preparedness against public health emergencies. This can involve monitoring healthcare facility capacities and the overall preparedness of the healthcare system to respond to an emergency [46].

Poor infrastructure, absence of emergency stockpiles, inadequate logistics, and shortages of medical supplies can be potential obstacles to achieving UHC during health emergencies [30, 35, 57, 58]. A strong public health infrastructure can help to ensure that healthcare resources are distributed equitably, based on need rather than ability to pay. This is particularly important during a pandemic, when resources may be scarce and demand for healthcare services is high [90]. Integrating pharmaceutical supply chain activities with modern technologies and establishing strong relationships between manufacturers, distributors, prescribers, and insurance organizations can help ensure that essential supplies and logistics are available promptly [91]. Efficient procurement and an effective supply chain management system are essential components of a well-functioning healthcare system. They can help ensure that essential medicines, medical supplies, and equipment are available where and when they are needed, which is critical to achieving UHC and providing quality healthcare services to all individuals, regardless of their ability to pay [92].

The main challenges for universal financial protection were inadequate healthcare funds [35]. Context specific health financing mechanisms are essential to provide strong and sustainable health financing and move towards UHC [44, 93]. Additionally, cross-subsidisation from rich to poor and low-risk to high-risk groups provide universal access for the entire population [94]. Similarly, reducing of health systems reliance on OOP payments and maximising risk pools were supportive to achieving UHC [60]. Universal health coverage can play a significant role in improving human security by providing financial protection against the cost of healthcare. In many countries, people face significant financial barriers to accessing healthcare services, and as a result, they may be forced to forgo necessary care or incur significant debt to pay for it. The example of Thailand is a good illustration of the potential benefits of UHC. Thailand implemented a comprehensive UHC program in the early 2000s, which provided coverage for all citizens and legal residents. Over the course of a decade, this program helped to reduce the annual impoverishment rate due to medical costs from 2.71% to 0.49% [61].

Poor leadership and absence of clear government policy led to fragmentation and poor health system response [35, 66]. It is essential to involve a diverse range of stakeholders in pandemic preparedness and response efforts to ensure that a comprehensive and effective response is implemented. Collaborative efforts that include input from various stakeholders are more likely to lead to successful outcomes in mitigating the impact of a pandemic [73, 95]. Good leadership is essential for effective outbreak response because it helps to coordinate and guide the efforts of different stakeholders, including health workers, community leaders, and government officials. A strong leader can help to build trust and confidence among the community, mobilise resources, and ensure that everyone is working together towards a common goal [54]. Health systems governance is essential for creating RHS that can respond to emerging health challenges, such as pandemics, as well as ongoing health concerns. By building strong partnerships and accountability mechanisms, health systems can better address the needs of individuals and communities, and improve overall health outcomes [48]. Cultivating bottom-up and top-down forms of accountability is also important to improve the quality and coverage of health services [96].

This study will provide an insight on RHS framework for achieving UHC and health security with an integrated and multi-sectoral approach that considers the health system building blocks and contextual factors. The limitations of this review include that the study did not quantitatively estimate the extent of resilient health system for UHC and health security. This is because we used articles based on various quantitative, qualitative, and mixed methods. In addition, we used the Rees et al. appraisal instrument as a guiding framework for the eligibility criteria [25]. The Rees specifies some methodological criteria for diverse types of studies but does not have a cut-off point for excluding studies.

This review provides evidence of the successes and challenges of RHS and its impact on achieving UHC globally. The review will also give an insight into the key determinants of RHS to achieve long term health sector goals. It will raise health programmers’ awareness of the importance of RHS and initiate an idea for future discussion and arguments on the subject. The review will also help policymakers and government officials to revise and update their strategic plans and policy directions. This review will also assist policy makers in introducing accountability within public institutions to provide more inclusive and equitable health services without excluding any population groups to achieve UHC. This review will help policymakers to formulate an agreed core set of global and national indicators to improve their health systems performance. This review will also help future researchers as baseline information.

## Conclusions

The aspiration for UHC and health security will be realised only through a RHS. Advanced healthcare infrastructures and adequate number of health care workers are essential to build a RHS; however, they are not adequate to protect the health systems from potential failures. The health system’s ideology, traditions in policymaking and management, orientation of service delivery, capacities, motivation, and morale of healthcare workers can affect the nation’s health system. Context-specific redistribution of health workers, task-shifting policy, and result-based health financing policy are helpful in building RHS. It is high time that countries transform their health systems through an integrated and multi-sectoral approach to serve as a road map to realise UHC and health security. We are also recommending for future research studies to focus on building a RHS that can support UHC and ensure health security. Such future studies shall be conducted at the national, regional and sub-regional levels to provide context-specific guidance.

### Supplementary Information

**Additional file 1.** Search strategy.

**Additional file 2.** ENTREQ checklist.

**Additional file 3.** PRISMA-ScR checklist.

## Data Availability

Not applicable.

## Abbreviations

ENTERQ: Enhancing transparency in reporting the synthesis of qualitative research
GHS: Global health security
HIS: Health information system
HRIS: Human resource information system
HS: Health system
MeSH: Medical subject headings
PROSPERO: Prospective register of systematic reviews
UHC: Universal health coverage
RHS: Resilient health system
SDGs: Sustainable development goals
WHO: World Health Organization
