# Supplementary material for: Building a resilient health system for universal health coverage and health security: a systematic review
Source: Glob Health Res Policy. 2024 Jan 4;9:2. doi: 10.1186/s41256-023-00340-z (PMC10765832; doi:10.1186/s41256-023-00340-z)
Supplement: Supplementary file 1 — Additional file 1. Search strategy. [file 41256_2023_340_MOESM1_ESM.docx]

PubMed search strategy

1. ((health system*[Title/Abstract] OR health service*[Title/Abstract] OR health financing[Title/Abstract] OR health workforce[Title/Abstract] OR health information[Title/Abstract] OR leadership[Title/Abstract] OR governance[Title/Abstract] OR medicine[Title/Abstract] OR diagnostics[Title/Abstract] OR infrastructure[Title/Abstract]) AND (resilien*[Title/Abstract] OR strong[Title/Abstract] OR preparedness[Title/Abstract] OR responsive[Title/Abstract] OR adaptive[Title/Abstract] OR transformative[Title/Abstract] OR absorptive[Title/Abstract])) AND (universal health coverage[Title/Abstract] OR universal coverage[Title/Abstract] OR universal access[Title/Abstract] OR equity[Title/Abstract] OR quality[Title/Abstract] OR financial protection[Title/Abstract])

2. ((health system*[Title/Abstract] OR health service[Title/Abstract] OR health financing[Title/Abstract] OR health workforce[Title/Abstract] OR health information[Title/Abstract] OR leadership[Title/Abstract] OR governance[Title/Abstract] OR medicine[Title/Abstract] OR diagnostics[Title/Abstract] OR infrastructure[Title/Abstract]) AND (resilien*[Title/Abstract] OR strong[Title/Abstract] OR preparedness[Title/Abstract] OR responsive[Title/Abstract] OR adaptive[Title/Abstract] OR transformative[Title/Abstract] OR absorptive[Title/Abstract])) AND (health security[Title/Abstract] OR prevention[Title/Abstract] OR detection[Title/Abstract] OR response[Title/Abstract])
