# Supplementary material for: Building a resilient health system for universal health coverage and health security: a systematic review
Source: Glob Health Res Policy. 2024 Jan 4;9:2. doi: 10.1186/s41256-023-00340-z (PMC10765832; doi:10.1186/s41256-023-00340-z)
Supplement: Supplementary file 2 — Additional file 2. ENTREQ checklist. [file 41256_2023_340_MOESM2_ESM.docx]

**ENTERQ checklist**

Enhancing transparency in reporting the synthesis of qualitative research: the ENTREQ statement

| **S. no** | **Item** | **Guide and description** | **Page** |
| --- | --- | --- | --- |
| 1 | Aim | State the research questions the synthesis addresses. | Page 4 lines 86-91 |
| 2 | Synthesis methodology | Identify the synthesis methodology or theoretical framework which underpins the synthesis, and describe the rationale for choice of methodology (e.g., meta-ethnography, thematic synthesis, critical interpretive synthesis, grounded theory synthesis, realist synthesis, meta-aggregation, meta-study, framework synthesis). | Page 5-6 lines 131-142 |
| 3 | Approach to searching | Indicate whether the search was pre-planned (comprehensive search strategies to seek all available studies) or iterative (to seek all available concepts until they theoretical saturation is achieved). | Page 4 lines 100-106 |
| 4 | Inclusion criteria | Specify the inclusion/exclusion criteria (e.g., in terms of population, language, year limits, type of publication, study type). | Page 5 lines 109-113 |
| 5 | Data sources | Describe the information sources used (e.g., electronic databases (MEDLINE, EMBASE, CINAHL, psycINFO, Econlit), grey literature databases (digital thesis, policy reports), relevant organisational websites, experts, information specialists, generic web searches (Google Scholar) hand searching, reference lists) and when the searches conducted; provide the rationale for using the data sources. | Page 4 lines 100-103 |
| 6 | Electronic Search strategy | Describe the literature search (e.g., provide electronic search strategies with population terms, clinical or health topic terms, experiential or social phenomena related terms, filters for qualitative research, and search limits). | Page 4 lines 102 -106 |
| 7 | Study screening methods | Describe the process of study screening and sifting (e.g., title, abstract and full text review, number of independent reviewers who screened studies). | Page 5 lines 117-121 |
| 8 | Study characteristics | Present the characteristics of the included studies (e.g., year of publication, country, population, number of participants, data collection, methodology, analysis, research questions). | Page 6 lines 146-151 |
| 9 | Study selection results | Identify the number of studies screened and provide reasons for study exclusion (e.g., for comprehensive searching, provide numbers of studies screened and reasons for exclusion indicated in a figure/flowchart; for iterative searching describe reasons for study exclusion and inclusion based on modifications the research question and/or contribution to theory development). | See Figure 1 |
| 10 | Rationale for appraisal | Describe the rationale and approach used to appraise the included studies or selected findings (e.g., assessment of conduct (validity and robustness), assessment of reporting (transparency), assessment of content and utility of the findings). | Page 5 lines 123-124 |
| 11 | Appraisal items | State the tools, frameworks and criteria used to appraise the studies or selected findings (e.g., Existing tools: CASP, QARI, COREQ, Mays and Pope; reviewer developed tools; describe the domains assessed: research team, study design, data analysis and interpretations, reporting). | Page 5 lines 123-124 |
| 12 | Appraisal process | Indicate whether the appraisal was conducted independently by more than one reviewer and if consensus was required. | Page 5 lines 123-124;  Page 15 lines 438-443 |
| 13 | Appraisal results | Present results of the quality assessment and indicate which articles, if any, were weighted/excluded based on the assessment and give the rationale. | Page 15 lines 438-443 |
| 14 | Data extraction | Indicate which sections of the primary studies were analysed and how were the data extracted from the primary studies? (e.g., all text under the headings “results /conclusions” were extracted electronically and entered into a computer software). | Page 5 lines 125-130 |
| 15 | Software | State the computer software used, if any. | N/A |
| 16 | Number of reviewers | Identify who was involved in coding and analysis. | Pages 5-6 lines 123-142 |
| 17 | Coding | Describe the process for coding of data (e.g., line by line coding to search for concepts). | See table 1 |
| 18 | Study comparison | Describe how were comparisons made within and across studies (e.g., subsequent studies were coded into pre-existing concepts, and new concepts were created when deemed necessary). | Pages 12-14 lines 342-435 |
| 19 | Derivation of themes | Explain whether the process of deriving the themes or constructs was inductive or deductive. | Pages 5-6 lines 131-142 |
| 20 | Quotations | Provide quotations from the primary studies to illustrate themes/constructs, and identify whether the quotations were participant quotations of the author’s interpretation. |  |
| 21 | Synthesis output | Present rich, compelling and useful results that go beyond a summary of the primary studies (e.g., new interpretation, models of evidence, conceptual models, analytical framework, development of a new theory or construct). | Pages 6-12 lines 159-340 |
